# Description de *Nosopsyllus (N.) Atsbi* n. sp. (Siphonaptera : Ceratophyllidae) d’Éthiopie et révision de l’espèce affine *N. (N.) Incisus* (Jordan & Rothschild, 1913) ; discussion biogéographique

**DOI:** 10.1051/parasite/2012191031

**Published:** 2012-02-15

**Authors:** J.-C. Beaucournu, Y. Meheretu, K. Welegerima, T. Mergey, A. Laudisoit

**Affiliations:** 1 Laboratoire de Parasitologie et Zoologie appliquée, Faculté de Médecine et Institut de Parasitologie de l’Ouest 2, avenue du Professeur Léon Bernard 35043 Rennes Cedex France; 2 Department of Biology, Mekelle University P.O. Box 3102 Mekelle Ethiopia; 3 Laboratoire de Parasitologie, Mycologie et Immunologie Parasitaire, CHRU 2, rue Henri Le Guilloux 35033 Rennes Cedex France; 4 Institute of Integrative Biology, The University of Liverpool, Biosciences Building Crown Street Liverpool L69 7ZB United Kingdom; 5 Evolutionary Ecology Group, University of Antwerp, Groenenborgerlaan 171 Antwerpen 2020 Belgique

**Keywords:** *Nosopsyllus atsbi* n. sp., *Nosopsyllus incisus incisus*, *Nosopsyllus incisus traubi* n. ssp., *Nosopsyllus incisus lewisi* n. ssp., Afrique orientale, biogéographie, *Nosopsyllus atsbi* n. sp., *Nosopsyllus incisus incisus*, *Nosopsyllus incisus traubi* n. ssp., *Nosopsyllus incisus lewisi* n. ssp., East Africa, biogeography

## Abstract

Nous décrivons un *Nosopsyllus* s. sto. nouveau du nord de l’Éthiopie, *N. atsbi*, montrant des ressemblances phylétiques avec *N. incisus* (Jordan & Rothschild, 1913), espèce cantonnée à la partie orientale de la région afrotropicale. Ceci nous conduit à revoir les populations classées comme *incisus* sur l’unique critère de la sétation du télomère (trois fortes soies marginales, au lieu des deux classiquement observées dans ces genre et sous-genre). Il apparaît que *N. incisus* s. sto. est connu au nord-est de la République Démocratique du Congo, au Kenya, au Burundi et en Tanzanie. Au nord et au sud de cette région (centre de l’Éthiopie, d’une part, Zambie et Malawi, d’autre part), deux taxa sont morphologiquement à part et nous les érigeons au rang de sous-espèces : *Nosopsyllus (N.) incisus traubi* n. ssp. et *N. (N.) incisus lewisi* n. ssp. À l’heure actuelle, le “complexe *incisus*” est riche de quatre taxa, à savoir, du nord au sud, *N. atsb*i n. sp., *N. incisus traubi* n. ssp., *N. incisus incisus* (Jordan & Rothschild, 1913) et *N. incisus lewisi* n. ssp.

## Abstract (en)

We describe a new *Nosopsyllus* s. sto., *N. atsbi*, from the north of Ethiopia, that shows phyletic similarities with *N. incisus* (Jordan & Rothschild, 1913), a species from the oriental part of the afrotropical region. This led us to reconsider the classification of the *incisus* populations based on the unique criteria of telomere’s setation (three robust marginal bristles, instead of the two typically present in members of this genus and subgenus). It appears that *N. incisus* s. sto. occurs in north-eastern Democratic Republic of the Congo, Kenya, Burundi and Tanzania. In the north and south of this region (respectively central Ethiopia, on one hand, Zambia and Malawi, on the other hand), two taxa are morphologically distinct and we erect them here to the rank of subspecies: *Nosopsyllus (N.) incisus traubi* n. ssp. and *N. (N.) incisus lewisi* n. ssp. Actually, four taxa are in “*incisus* complex”, namely: *N. atsbi* n. sp., *N. incisus traubi* n. ssp., *N. incisus incisus* (Jordan & Rothschild, 1913), and *N. incisus lewisi* n. ssp.

## Introduction

La seule révision du genre *Nosopsyllus* Jordan, 1966 (Siphonaptera : Ceratophyllidae) sur un plan géographique est celle de Lewis (1967) qui axe son article (ce devait être le premier d’une série, mais ce fut le seul à être publié) sur les “*African species*”. Ce titre est trompeur, on pourrait s’attendre à y trouver une étude sur les taxa afrotropicaux de ce genre. En fait, Lewis cible son propos sur les *Nosopsyllus* signalés sur le continent africain et cite, par exemple, *Nosopsyllus (N.) atlantis* Jordan, 1937, espèce paléarctique méridionale, endémique de la dorsale rocheuse marocaine avec son hôte, l’écureuil de Gétulie (*Atlantoxerus getulus*). De fait, un seul taxon dans ce genre, et sous-genre, répondait alors au critère “afrotropical”, *N. (N.) incisus* (Jordan & Rothschild, 1913), mais il est vrai que *N. fasciatus* et, à un moindre degré, *N. l. londiniensis*, primitivement paléarctiques, sont secondairement devenus cosmopolites, car dispersés avec les rats (*Rattus* spp.) et la souris (*Mus domesticus*), par voie maritime essentiellement. En région africaine, ces espèces restent cantonnées aux zones littorales où elles ne sont que peu représentées; elles manquent totalement dans la sous-région malgache (Beaucournu & Fontenille, 1993).

**Figure 1.**
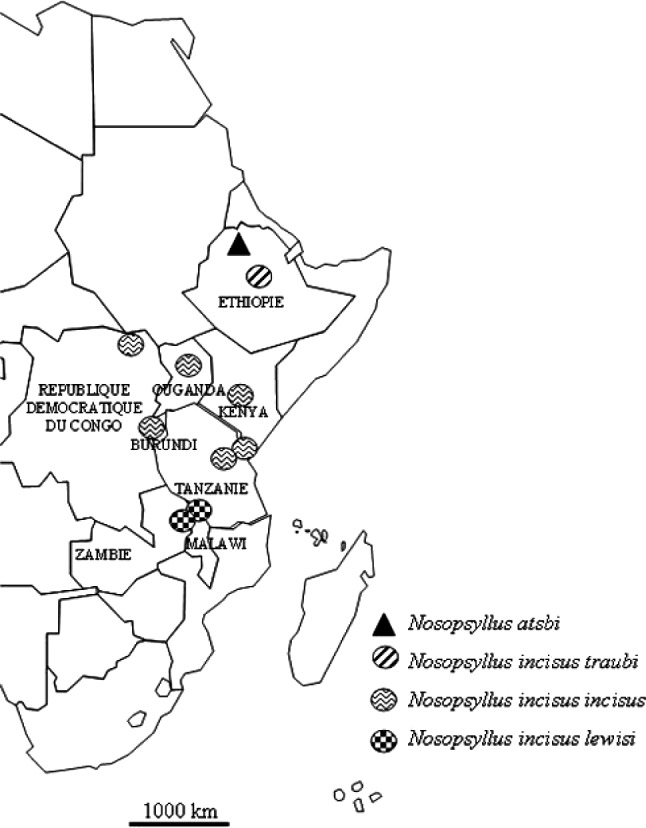
Moitié orientale de l’Afrique montrant la dispersion du “complexe *incisus*” : *Nosopsyllus atsbi* (nord de l’Éthiopie); *N. incisus traubi* (centre de l’Éthiopie); *N. incisus incisus* (J. & R.) (République Démocratique du Congo, Kenya, Ouganda, Burundi, Tanzanie); *N. incisus lewisi* (Zambie et Malawi, ces deux localisations étant, semble-t-il, très proches, voire contiguës).

De récentes prospections dans le nord de l’Éthiopie, ayant pour premier objectif de mieux connaître les rongeurs nuisibles aux cultures, puis secondairement leurs ectoparasites (Meheretu *et al.*, in press) nous ont livré une puce nouvelle que nous décrivons ici, *N. (N.) atsbi* n. sp. Son étude montre que, bien que constituant un taxon autonome, elle est apparentée à *N. (N.) incisus* (Jordan & Rothschild, 1913), taxon qui dès sa description, sous le nom de *Ceratophyllus incisus*, montra un destin intéressant et fut servi par la chance. Il fut récolté en “British East Africa” et décrit par Jordan & Rothschild en 1913, sur une série de femelles, dont l’holotype (il faudra attendre le travail de Lewis (1990) pour que le Kenya soit nommément désigné comme *terra typica*). Toutefois, ce matériel qui provient pour la majorité des “Aberdare Mts” contient également une femelle de “Buhamba, Congo” (*recte* Congo belge à cette époque). Le caractère discriminant essentiel est la présence, exceptionnelle dans ce sous-genre, d’un sinus sur le sternite VII. Pour le reste, l’espèce est, très justement, donnée comme voisine de *Ceratophyllus fasciatus*, puce paléarctique, secondairement implantée dans de nombreuses régions du globe par le fait de l’homme. En 1933, Jordan érige le groupe d’espèces auquel appartiennent *fasciatus* et *incisus* en un genre nouveau, le genre *Nosopsyllus*. Berteaux (1947) découvre le mâle de *N. incisus* à Gabu, région de Blukwa, au Congo belge (soit le Zaïre, devenu République Démocratique du Congo), mâle qu’il décrit sur les conseils de Jordan, celui-ci ayant vu ce matériel. Ce mâle, dont la description est assez sommaire, n’est à aucun moment considéré comme “neallotype”. De même que pour la femelle, il se distingue immédiatement des autres *Nosopsyllus* décrits par la présence, cette fois, de trois soies marginales sur le télomère, au lieu des deux classiques. Approximativement, 800 kilomètres séparent l’emplacement de récolte du matériel-type femelle provenant du Kenya de celui du mâle (*cf*. Figure 1).

Par la suite, *N. incisus* est cité de l’Ouganda par Hopkins (1947), du Tanganyika (Tanzanie) par Hubbard (1963) et du Burundi par Guiguen *et al.* (1980). Des articles plus ou moins généraux (Haeselbarth, 1966; Lewis, 1967; Smit, 1975; Traub *et al.*, 1983; Beaucournu, 2004) permettent de donner la répartition suivante, du nord au sud : Éthiopie, nord-est de la République Démocratique du Congo, Kenya, Ouganda, Burundi, Tanzanie, Zambie et Malawi.

La seule révision de *N. incisus* a été faite par Lewis (*op. cit.*). L’auteur remarque la variabilité de ce taxon et note “*there is some evidence for a southern subspecies. At present insufficient material precludes proper évaluation*”. Il faut noter que cet auteur ne pouvait connaître, à cette époque, la présence de cette puce en Éthiopie, ceci n’ayant été signalé qu’en 1983 par Traub *et al.* La récolte, en Éthiopie précisément, mais dans le nord de ce pays, d’une espèce nouvelle de *Nosopsyllus*, espèce rappelant *N. incisus* par certains caractères (essentiellement la présence de trois soies fortes sur le télomère), mais localisée au nord de la répartition connue de *N. incisus*, nous a incités à tenter une révision des taxa affines. Lorsque nous envisagerons l’ensemble de ces *Nosopsyllus*, nous utiliserons le terme de “complexe *incisus*”, ce terme réunissant les espèces *atsbi* et *incisus* s. l.

## Description

### *Nosopsyllus (Nosopsyllus) atsbi* Beaucournu, Meheretu & Laudisoit n. sp.

#### Matériel de description

Holotype ♂ sur *Arvicanthis dembeensis* (Rüppel, 1824) (= *A. niloticus* (Desmarest, 1822) *pro parte*, *in* Musser & Carleton, 1993), n° 1789, 1er janvier 2010; allotype ♀, deux ♂ et deux ♀ paratypes, sur le même hôte, mais n° 1853, 10 mars 2010; six ♂ et deux ♀ paratypes sur le même hôte, mais n° 1749, avril 2010; une ♀ paratype sur le même hôte, mais n° 1751, avril 2010; un ♂ paratype, sur le même hôte, mais n° 1982, 8 juillet 2010; un ♂ et une ♀ paratypes sur *Mastomys awashensis* Lavrenchenko, Likhnova & Baskevitch, 1998, n° 1750, avril 2010; un ♂ paratype, sur le même hôte, n° 1777, avril 2010, tous collectés à Golegole Naele, près d’Atsbi (Éthiopie), 13° 52’ N – 39° 43’ E, région du Tigray, à des altitudes supérieures à 2 300 m. Holotype, allotype et quelques paratypes sont dans les collections de J.-C. Beaucournu, ultérieurement déposées au Laboratoire d’Entomologie du Muséum national d’Histoire naturelle de Paris (France); les autres paratypes sont partagés entre les départements de biologie des Universités d’Anvers (Belgique) et de Mekelle (Éthiopie), le British Museum of Natural History (T. Howard – UK) et le Carnegie Museum of Natural History, Pittsburgh (J. Rawlins – USA).

*Derivatio nominis* de “Atsbi” : nom de la région de collecte, nom mis en apposition.

#### Description

Mâle évoquant *N. incisus* (J. & R.) par son télomère, s’en séparant aisément par son basimère et le bras apical du sternite IX; la femelle ne montre pas le profil du sternite VII ayant donné son nom à l’espèce (*incisus*), elle évoquerait davantage, sous cet aspect, un *N. (Gerbillophilus)* sp.

Capsule céphalique : elle est, comme classiquement dans ce genre, très arrondie, avec un petit tubercule frontal situé plus ou moins au tiers ventral; il est quelquefois à peine perceptible. OEil rond ou ovalaire en fonction de la mise au point, toujours bien pigmenté. Palpe labial à peu près aussi long que la moitié de la longueur de la coxa I; palpe maxillaire aussi long que cette coxa. Trois à quatre soies occipitales, la première peu développée. Trois grandes soies pré-oculaires, précédées de trois assez courtes.

Prothorax portant une rangée de cinq soies longues et érigées précédant une cténidie de 11 dents par côté. Mésothorax montrant quatre ou cinq soies érigées, précédées de cinq soies plus petites; présence de six *pseudo-setae* de chaque côté. Métathorax : cinq soies érigées, précédées par six plus petites; présence d’une spinule, peu pigmentée, en position dorsale. Patte III : la plus longue soie du tibia n’atteint pas l’apex du premier segment tarsal. Soies plantaires du distitarsomère au nombre de 15 à 16 chez le mâle, de 17 à 25 chez la femelle

Abdomen (segments non génitaux) : spinules présentes sur les segments I à VII, au nombre de 2-2-1-1-0 chez le mâle, 1-2-0-0-0 chez la femelle. Sternites II à VII portant, chez le mâle, 1-2-2-3-3-3 soies latérales, et chez la femelle 1 (plus 3 à 5 petites)-4 ou 5-5-4-5 ou 6-7 soies latérales.

**Figure 2.**
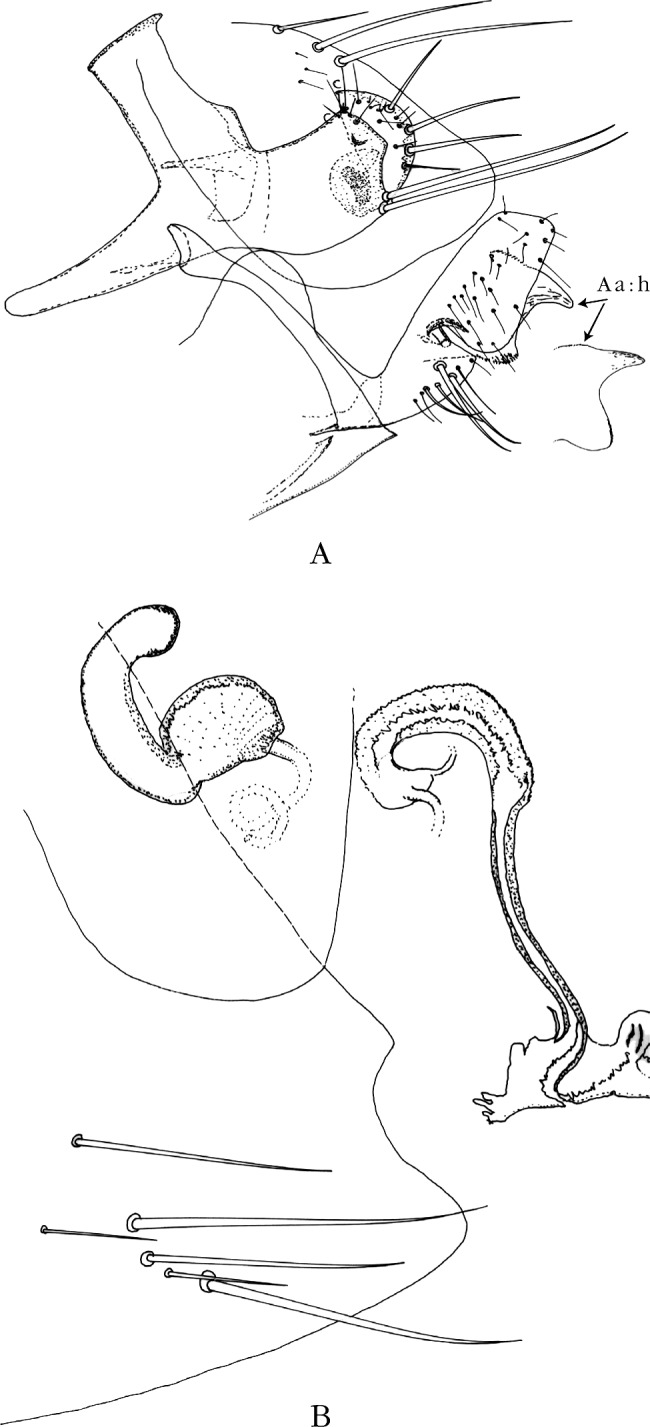
A–B. *Nosopsyllus (N.) atsbi* n. sp. A : Holotype, sternite VII, segments VIII et IX; A a, paratype, *hamulus* (h). B : Allotype, segment VII (*pro parte*), spermathèque et *ducti*.

Abdomen (segments génitaux du mâle) (Figure 2A). Tergite VIII petit, oblong, portant trois longues soies marginales et deux médianes; sternite VII régressé comme il est de règle. Le tergite IX montre un basimère quadrangulaire trapu, non triangulaire, portant deux longues soies à sa base. Télomère à bord antérieur droit, alors que le bord postérieur est très convexe et porte trois grosses soies marginales (comme chez *N. incisus*) et non deux; sternite IX : la partie basale du bras apical montre deux longues soies (comme chez *N. atlantis*) et non deux courtes.

Phallosome classique des *Nosopsyllus* s. sto. (*cf*. Traub *et al.*, 1983, planche 56 pour *N. fasciatus*, espèce avec laquelle nous ne voyons pas de différence constante). Toutefois l’*hamulus* est unique par sa forme en “bissac” (Figure 2A, h), montrant un “bec” dans la partie supérieure et, après un rétrécissement médian, s’élargissant dans sa partie inférieure (comparer avec les Figures 4A ou 4B); cet aspect n’est pas sans évoquer quelque peu celui de *N. durii* Hubbard, 1956 espèce décrite d’Iraq, puis retrouvée en Syrie, au Liban, en Grèce et en Turquie, où elle parasite les Arvicolidés et les Muridés (Orhan & Beaucournu, 1982).

Abdomen (segments génitaux de la femelle) (Figure 2B). Sternite VII de contour assez variable mais ne montrant jamais l’incisure classique des types de *N. incisus*. Spermathèque montrant une *bulga* subsphérique et une *hilla* très recourbée, ces deux caractères étant ceux des *Nosopsyllus* s. sto.; une ébauche de *papilla* est visible chez quelques exemplaires; la *bursa copulatrix* ne montre que trois quart de circonvolution, le *ductus spermathecae* est très long, le *ductus obturatus* est bien développé, mais ne peut être mesuré. Dimensions : mâles de 2,0 à 2,6 mm (moyenne 2,3; holotype 2,6); femelles de 3,2 à 3,8 mm (moyenne 3,48; allotype 3,5). Ces dimensions sont supérieures à la normale pour les *Nosopsyllus* s. sto.

#### Discussion

Sans tenir compte de sa taille, *N. atsbi* n. sp. ne peut être confondu avec aucun autre taxon du genre *Nosopsyllus*. Nous avons signalé que le télomère le rapprochait de *N. incisus*, mais cette ressemblance, si elle permet un rapprochement phylétique, est insuffisante pour ramener cette espèce à un rang subspécifique. Pour les autres caractères, on pourrait évoquer tantôt un *Gerbillophilus* (taille, forme du basimère, contour du sternite VII de la femelle), tantôt un autre *Nosopsyllus* s. sto.

## Révision du “complexe *incisus*”

### Matériel d’étude

Tous les Siphonaptères du genre et sous-genre *Nosopsyllus* à notre disposition, montrant chez le mâle trois soies marginales sur le télomère, seront examinés, ceci impliquant la prise en compte de *N. atsbi*. Nous citerons les spécimens étudiés en les classant approximativement du nord au sud et d’ouest en est. Un dessin du tergite VIII et du segment IX sera donné pour un mâle de chaque lot, de même que le contour (et la sétation) du sternite VII et le *ductus bursae* pour les femelles : ces indications sont reportées dans la liste ci-dessous. Nous rappelons que deux espèces monotypiques étaient concernées, *N. incisus* et *N. atsbi*, lorsque nous avons entrepris ce travail. La majorité des préparations qui nous ont été confiées donnent l’altitude en pieds anglo-saxons (soit 0,3048 m pour 1 pied). Nous avons fait la correction brute sans tenter d’arrondir ce chiffre, d’où la pseudo-précision des données obtenues.

### Éthiopie

**Figure 3.**
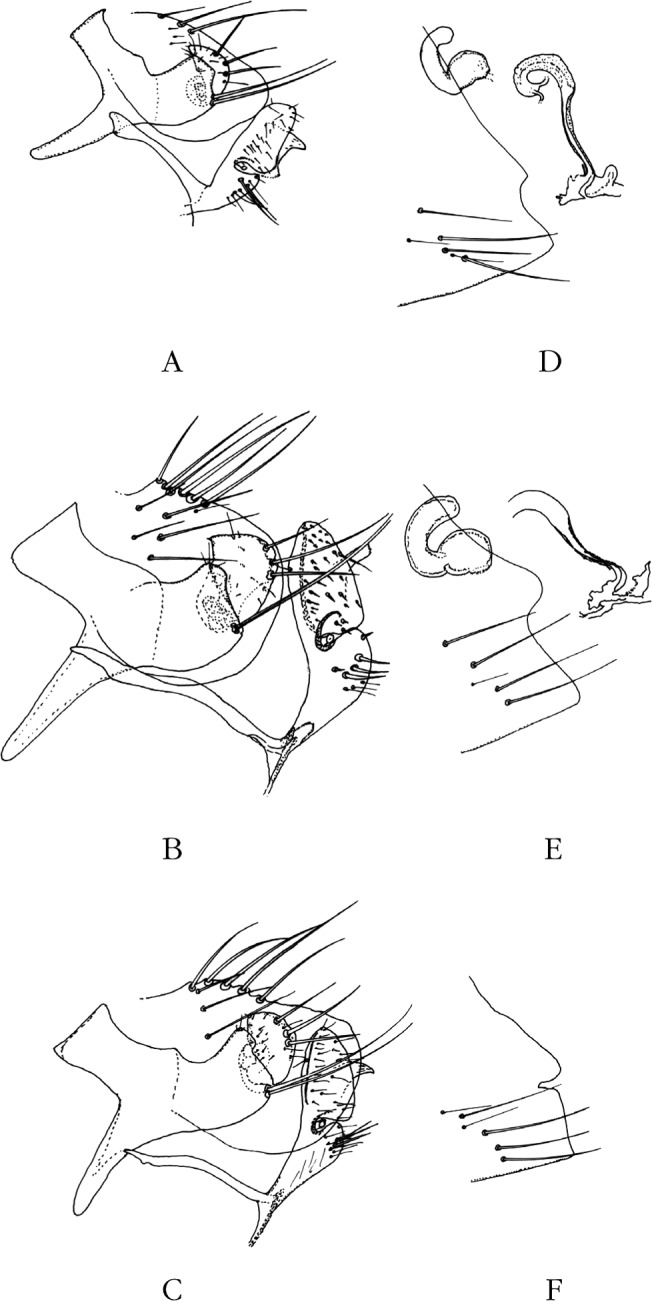
A–C. Tergite VIII et segment IX & Figures 3D-F. – Sternite VII et, éventuellement, spermathèque et *ductus bursae* de *Nosopsyllus atsbi*, Éthiopie, Atsbi (A et D), de *N. incisus traubi* n. ssp., Éthiopie, Bale (B et E), et de *N. incisus incisus*, Ouganda (C et F).

Province du Tigray, dix ♂, six ♀ (dont l’holotype (Figures 2A, 3A) et l’allotype (Figures 2B, 3D) de *N. atsbi* B., M. & L., les autres étant des paratypes) sur *Arvicanthis dembeensis*, Atsbi, district d’Atsbi Wonberta (13° 38’ 54’’ N – 39° 10’ 25’’ E), alt. 2 630 m, mars à juillet 2010; deux ♂, une ♀ (paratypes) sur *Mastomys awashensis*, même endroit, avril 2010 (Meheretu *et al. rec.*) (*pro parte*, *in* : Coll. J.-C. B.). Province de Bale, sur *Dendromus* sp., un ♂ (partiellement disséqué) (Figure 3B), une ♀, Dinshu (6° 46’ N – 39° 40’ E), Parc national, Monts Bale, 9 900 pieds (3 017 m), 21 février 1973; *ex nido Dendromus*, quatre ♂ (dont trois partiellement disséqués) (Figure 6), trois ♀ (Figure 3E), même endroit, même date; *ex nido* rongeur/oiseau, deux ♂, deux ♀, même endroit, même date (R. Traub & J. Ash *rec.*) (*in* : Coll. Carnegie Museum of Natural History – CMNH).

### République Démocratique du Congo

Province de l’Ituri : aucun spécimen n’a pu être examiné. Beaucournu & Rahm (1978) ont étudié 1 360 puces de rongeurs et petits insectivores dans la province du Kivu, mais aucun *Nosopsyllus* n’y a été collecté. Nous nous appuierons donc sur le dessin de Berteaux (1947).

### Ouganda

Province de Kigizi (1° 00’ S – 29° 45’ E), Kanaba, 10 septembre 1940, sur *Grammomys surdaster*, un ♂ (Figure 3C); même endroit, même hôte, 29 septembre 1940, une ♀ (Figure 3F) (G.H.E. Hopkins *rec.*) (*in* : Coll. British Museum of Natural History – BMNH).

### Kenya

**Figure 4.**
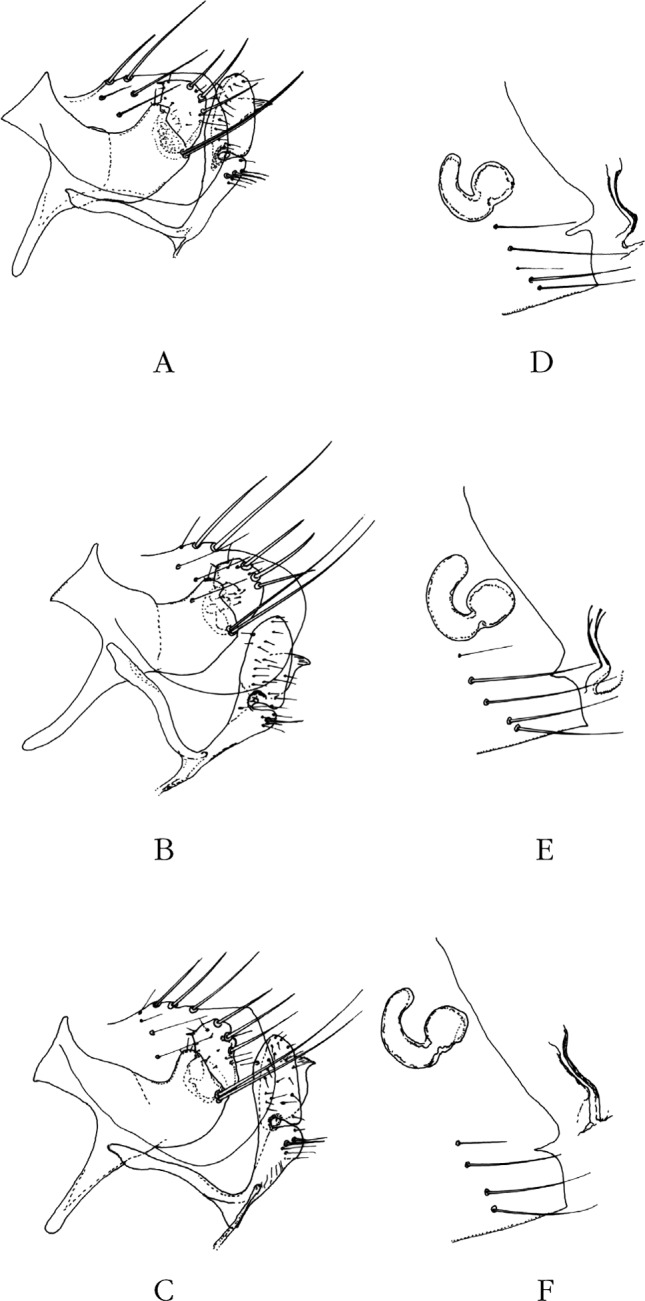
A–C. Tergite VIII et segment IX & Figures 4D-F. – Sternite VII, spermathèque et *ductus bursae* de *Nosopsyllus incisus incisus*, Kenya (A et D), de *N. i. incisus*, Burundi (B et E), et de *N. i. incisus*, Tanzanie (Sunga) (C et F).

Province de la Vallée du Rift, district de Nakuru (0° 17’ S – 36° 04’ E), alt. 7 000 pieds (2 133 m), nom de la localité illisible, 4 juillet 1943, sur *Thamnomys ibeanus*, deux ♂ (Figure 4A), une ♀ (Figure 4D) (H. Hoogstraal *rec.*) (*in* : Coll. CMNH).

### Burundi

Province de Bururi (3° 57’ S – 29° 30’ E), région de Tora, alt. *ca*. 2 000 m, de décembre 1977 à juin 1978, sur *Oenomys hypoxanthus* 27 ♂♀ (Figures 4B, 4E), sur cinq autres espèces de rongeurs (*Lophuromys sikapusi*, *Otomys denti*, *Mastomys natalensis*, *Praomys jacksoni* et *Mylomys dybowskii*) 14 ♂♀ (C. Guiguen & J. Vissault *rec.*) (*in* : Coll. J.-C. B.).

### Tanzanie (anciennement Tanganyika)

**Figure 5.**
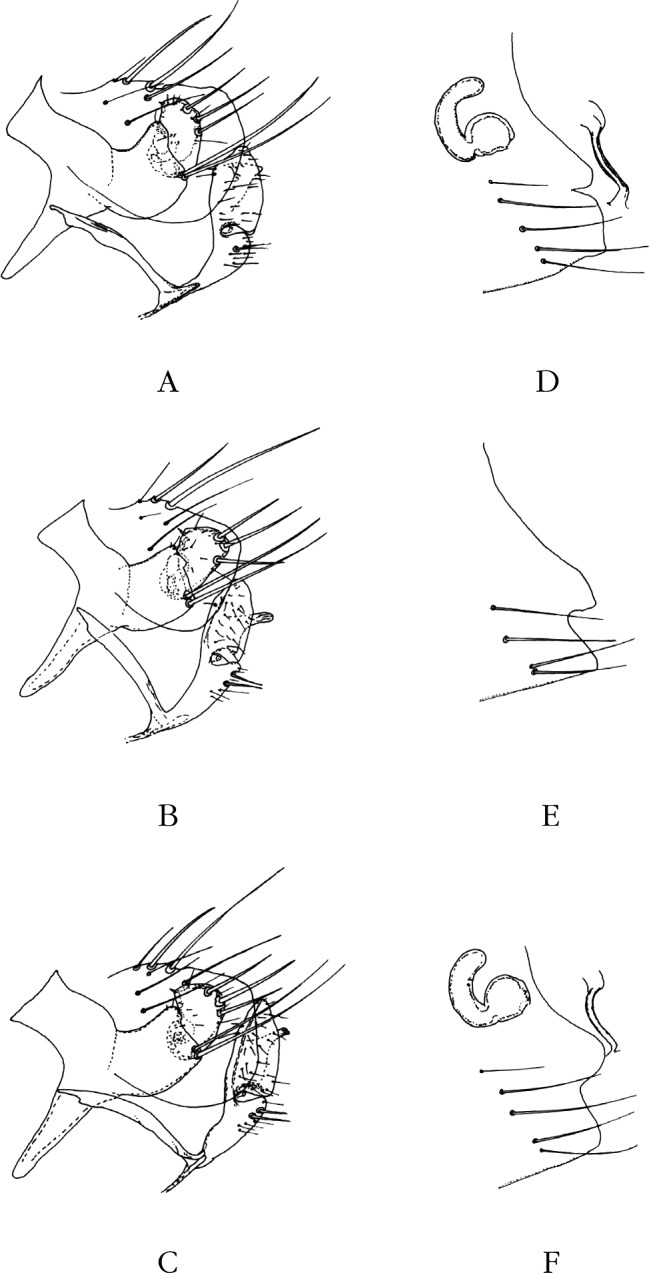
A–C. Tergite VIII et segment IX & Figures 5D-F. – Sternite VII et, éventuellement, spermathèque et *ductus bursae* de *Nosopsyllus incisus incisus*, Tanzanie (Berega) (A et D), de *N. incisus lewisi* n. ssp., Malawi (B et E), et de *N. incisus lewisi* n. ssp., Zambie (C et F).

Province de Tanga, district de Lushoto (4° 47’ S – 38° 17’ E), novembre 1963, sur *Lophuromys f. margarettae* (= *L. flavopunctus*), un ♂, deux ♀; même endroit, octobre 1963, sur *Thamnomys* (*recte Grammomys*) *dolichurus surdaster*, une ♀ (C.A. Hubbard *rec.*) (*in* : Coll. CMNH); Lushoto, Western Usambara Mts, alt. 4 500 pieds (1 371 m), 27 octobre 1962, sur *Grammomys dolichurus surdaster*, une ♀ (Figure 4F); même endroit, 8 janvier 1963, sur *Lophuromys* sp., un ♂; Sunga, Western Usambara Mts, alt. 6 500 pieds (1 981 m), 20-25 novembre 1962, sur *Thamnomys* (*recte Grammomys*) *dolichurus surdaster*, un ♂ (Figure 4C) et *Lophuromys f. margarettae* (= *L. flavopunctus*), quatre ♂, une ♀ (*in* : Coll. CMNH); Berega, (6° 14’ S – 37° 09’ E), alt. 749 m, sans date, sur *Grammomys* sp., trois ♂ (Figure 5A), deux ♀; Batai, Magamba, 29 janvier 2006, sur *Lophuromys kilonzoi* une ♀ (Figure 5D) (A. Laudisoit *rec.*) (*pro partei*, *in* : Coll. J.-C. B. et A. L.).

### Zambie

Nyika Plateau (10° 34’ S – 33° 42’ E), 12 décembre 1963, sur *Aethomys* sp., une ♀ (Figure 5F); même endroit, 17 décembre 1963, même hôte, un ♂, une ♀; même endroit, 30 décembre 1963, sur *Pelomys* sp., une ♀ (Figure 5C) (I.F. Keymer *rec.*) (*in* : Coll. BMNH).

### Malawi (= North Rhodesia)

Nyasaland, Nyika Plateau, 12 juin 1962, sur *Grammomys cometes*, une ♀ (J.F. Hanney *rec.*); Nyika Plateau (10° 36’ S – 33° 45’ E), 7 300 pieds (2 225 m), 30 août 1962, sur *Grammomys dolichurus surdaster*, une ♀ (Figure 5E); même endroit, 20 août 1962, même hôte, une ♀; même endroit, 29 août 1962, sur *Dendromus mesomelas*, un ♂, une ♀ (J.M. Ingles *rec.*); Nyika Plateau (10° 40’ S – 33° 37’ E), 14 décembre 1963, sur *Aethomys* sp., deux ♂ (Figures 5B, 7) (I.F. Keymer *rec.*) (*in* : Coll. BMNH).

## Étude morphologique

Caractères généraux (non sexuels) : le palpe labial et le palpe maxillaire sont pratiquement de même longueur que la *coxa*, sauf chez *N. atsbi* chez qui le palpe labial est nettement plus court que le palpe maxillaire. L’oeil est normalement déve- loppé et pigmenté. Le prothorax, le mésothorax et le métathorax, de même que les deux ou trois premiers tergites montrent des soies érigées dorsalement, ce caractère étant plus net chez les mâles. La majorité des autres *Nosopsyllus* s. sto. ont ce type de soies jusqu’au tergite VII. Des spinules sont présentes, sur les tergites I-IV, selon le mode moyen 2-2-2-1 (plus rarement 3- 2-2-1 ou 1-1-1-0).

Soies de la patte III. Chez les mâles, la plus longue soie du tibia atteint la moitié, voire les deux tiers, du tarse I; la plus longue soie du tarse I est plus variable, sa longueur va de la base du tarse III, à la base du tarse IV; enfin, la plus longue soie du tarse II atteint toujours le distitarsomère, au moins sa base, souvent la moitié de sa longueur, ou même son apex. Pour ces divers caractères, nous ne voyons aucun élément en faveur d’une spéciation ou même d’une subspéciation.

**Figure 6.**
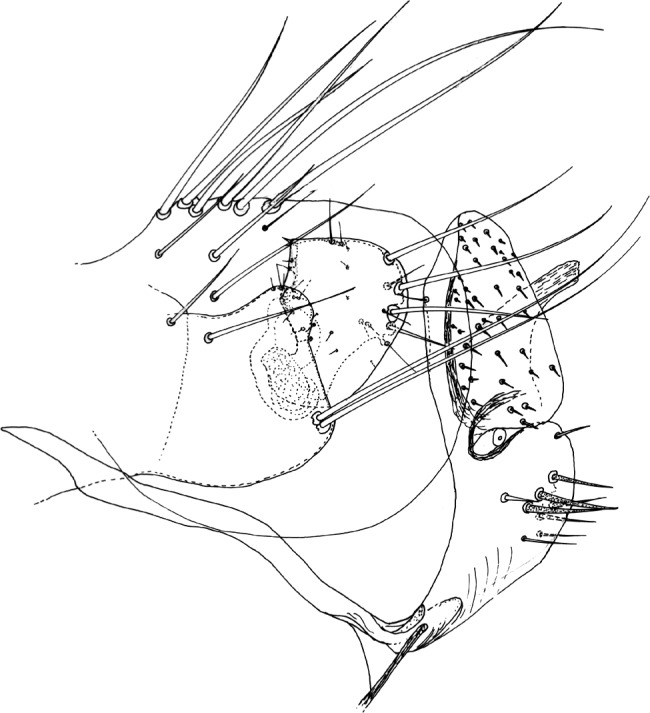
*Nosopsyllus (N.) incisus traubi* n. ssp. Tergite VIII et segment IX du ♂ holotype sur *Dendromus* sp., Dinshu, Parc National Mts Bale (Éthiopie), 3 017 m, 21 février 1973 (R. Traub & J. Ash *rec.*).

**Figure 7.**
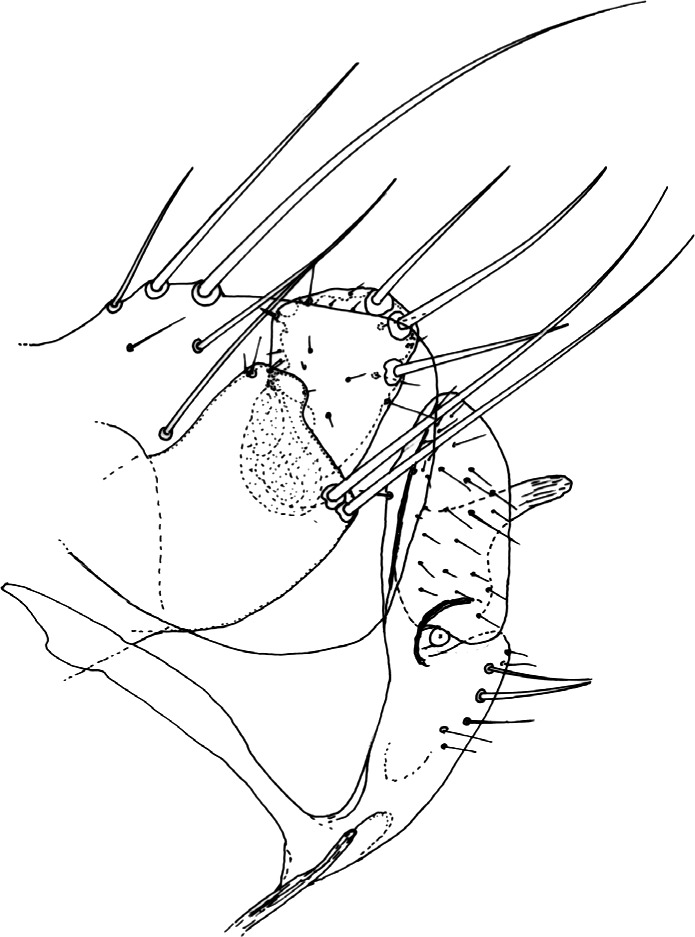
*Nosopsyllus (N.) incisus lewisi* n. ssp. Tergite VIII et segment IX du ♂ holotype sur *Aethomys* sp., Nyika Plateau (Malawi), *ca* 2 220 m, 14 décembre 1963 (I.F. Keymer *rec.*).

Segments génitaux mâles. Le tergite VIII est allongé et peu haut chez *N. atsbi* (Figures 2A, 3A) (comme celui de *N. londiniensis*). Dans tous les autres cas, il est grand : grand et arrondi chez les exemplaires examinés d’Éthiopie, province de Bale (Figures 3B, 6), mais aussi du Kenya (Figure 4A), du Burundi (Figure 4B) et *pro parte* de Tanzanie (Berega) (Figure 5A); grand mais anguleux à l’apex chez les exemplaires de Tanzanie (Sunga) (Figure 4C), du Malawi (Figures 5B, 7) et de Zambie (Figure 5C); le seul exemplaire étudié provenant de l’Ouganda est intermédiaire entre ces deux formes (Figure 3C).

Tergite IX. Manubrium : doucement courbé vers le haut chez *N. atsbi*, doucement courbé vers le bas ou rectiligne chez les autres. Le lobe dorsal du basimère est, dorsalement, plat ou arrondi. Il est plat et allongé chez *N. atsbi*, plat et court chez le ou les exemplaires de l’Ouganda, du Kenya, du Burundi et du Malawi; il est arrondi en Tanzanie (Sunga et Berega) et en Zambie; les exemplaires de Bale sont intermédiaires entre ces deux formes.

L’analyse du télomère est complexe, que l’on cherche des différences dans sa forme ou dans l’implantation des trois soies marginales, celles-ci étant toujours placées dans la moitié supérieure (la plus basse est quelquefois exactement médiane). Le bord distal est soit:

- en demi-croissant de lune : Éthiopie (Atsbi);
- arrondi avec un bord libre long entre la petite soie antéro-marginale et la première des fortes soies marginales (c’est-à-dire plus long ou aussi long que la distance séparant la zone d’implantation de ces trois soies) : Éthiopie (Bale);
- arrondi avec un bord libre court : Ouganda, Kenya, Burundi et peut-être Zambie;
- plus ou moins droit : Tanzanie (Sunga et Berega);
- le télomère est, dans son ensemble, plus ou moins triangulaire : Malawi et Zambie.

Ce caractère semble avoir une valeur discriminante.

Sternite IX. Un petit caractère se montre d’emblée au niveau de la tête du bras proximal du sternite : celle-ci est plutôt courte et trapue chez *N. atsbi*, elle est longue et étroite chez les autres exemplaires.

La partie basale du bras distal porte :

- deux ou trois soies longues : Éthiopie (Atsbi);
- deux ou trois soies courtes, trapues, sclérotisées; les autres petites soies sont également trapues : Éthiopie (Bale);
- deux, rarement une, soies courtes, trapues mais peu sclérotisées, chez les autres exemplaires.

La partie distale de ce bras est également intéressante. Chez une population (Éthiopie, Bale), le lobe dorsal est couvert de micro-épines doucement sclérotisées; ce sont des micro-soies transparentes chez les autres. L’apex de ce bras est soit coupé assez abruptement (Éthiopie, Atsbi), soit nettement arrondi et symétrique (Kenya), soit ogival et asymétrique (pratiquement toutes les autres origines). Aucune dichotomie ne semble ressortir de ces morphologies.

Phallosome : sa structure est, dans l’ensemble proche de celle de *N. fasciatus*. Nous n’avons pu mettre en évidence de différences stables dans notre matériel; même *N. atsbi* ne peut être différencié de *N. incisus* par cet organe. Toutefois, l’*hamulus* est de forme variée suivant les populations. En “bec” trapu dans l’Ouganda, le Burundi et la Tanzanie; en “bec” plus allongé en République Démocratique du Congo (*teste* Berteaux, 1947) et au Kenya; en processus à bords parallèles, beaucoup plus long que haut, en Éthiopie (Bale), au Malawi et en Zambie. L’*hamulus* de *N. atsbi* n. sp. est, nous l’avons signalé, unique dans ce complexe par sa forme en “bissac”, la partie ventrale étant de nouveau convexe.

Segments génitaux femelles. Le sternite VII est très variable. Le type *incisus* ne se rencontre qu’en Ouganda (Figure 3F), au Kenya (d’où viennent les types !) (Figure 4D) et dans nos populations de Tanzanie (Figures 4F, 5D). C’est sans aucun doute le cas des femelles accompagnant le mâle décrit par Berteaux (*op. cit.*) car, sans cela, le lien avec *N. incisus* (*sensu* J. & R.) n’aurait pu être établi. Celles du Burundi en sont proches, mais l’incisure s’est ouverte en un large “V” (Figure 4E). Pour les exemplaires d’Éthiopie (Atsbi (Figures 2B, 3D) et Bale (Figure 3E)), du Malawi (Figure 5E) et de Zambie (Figure 5F), il ne s’agit plus que d’un lobe, plus ou moins arrondi, surplombant une concavité subventrale. La sétation de ce sternite est toujours de quatre longues soies; s’y ajoutent, fortuitement semble-t-il, une ou deux soies accessoires, plus petites.

Spermathèque : elle montre une forme classique chez *Nosopsyllus* : *bulga* pratiquement sphérique et *hilla* en crosse de canne. Plusieurs exemplaires (Ouganda, Malawi) ont une spermathèque soit déformée, soit mal orientée, ce qui nous prive d’éléments de comparaison. La zone d’insertion du *ductus spermathecae* montre des variations, mais le faible effectif de femelles examinées rend toute conclusion aléatoire. Enfin, la longueur du *ductus bursae* (en ne prenant en compte que la partie sclérifiée, facile à mesurer), par rapport à une autre dimension, par exemple un segment du tarse III, ne nous a rien donné.

## Discussion

Les hôtes du “complexe *incisus*” sont les Muridae s. sto., rongeurs ubiquistes, présents en particulier dans les régions paléarctique, afrotropicale, orientale et malaise. Notre tentative d’analyse du “complexe *incisus*”, c’est-à-dire de toutes les espèces ayant trois soies fortes sur la marge distale du télomère, montre que *N. atsbi* B., M. & L. fait figure d’espèce souche pour tout le complexe. Il faut ici rappeler que le genre *Nosopsyllus*, largement répandu en régions palaéarctique et orientale, manquerait à la région afrotropicale, sous-région malgache incluse, en l’absence du “complexe *incisus*”. Sur le continent africain, les Ceratophyllidae sont rares : les Amphipsyllinae manquent totalement, les Ceratophyllinae sont seulement représentés, en dehors de ce complexe, par le genre endémique *Libyastus*, inféodé aux Sciuridae; quant aux Leptopsyllinae, ils ne sont présents en Afrique continentale qu’avec *Leptopsylla aethiopica* polytypique, endémique, parasite de Muridae; le genre *Leptopsylla* est, lui, connu des régions paléarctique et surtout orientale. Si *Lybiastus* est connu de l’Afrique orientale comme de l’Afrique occidentale, *Leptopsylla aethiopica*, comme le “complexe *incisus*”, n’est signalé que dans l’est africain. Certes, l’origine laurasienne des Ceratophyllidae peut, en partie, justifier cette “lacune”, mais d’autres facteurs sont certainement en cause. On ne peut retenir un argument climatique, car la richesse de cette famille en région orientale, y inclus la sousrégion wallacéenne, serait là pour nous contredire.

Ainsi que plusieurs auteurs l’ont souligné (Lewis, 1967; Haeselbarth, 1966; Traub *et al.*, 1983), *N. incisus*, ou dans l’optique de ce travail le “complexe *incisus*”, ne se rencontre qu’en altitude, généralement au-dessus de 2 000 m (l’altitude moyenne de collecte des exemplaires étudiés ici est de 2 000 m, avec une station à plus de 3 000 m, mais deux à moins de 800 m). Ce fait a eu comme conséquence de morceler, plus ou moins, l’aire de répartition de ces Siphonaptères et peut expliquer la variabilité observée (Carte 1).

Une première conclusion s’impose : c’est la validité de *N. (N.) atsbi* B., M. & L. (Éthiopie, province du Tigray). Une autre population (elle provient également d’Éthiopie, province de Bale) se démarque assez facilement, nous y reviendrons. De même, mais plus discrètement, les populations du Malawi et de Zambie se séparent des *N. (N.) incisus* s. sto. par la forme du télomère.

Curieusement, *N. incisus*, *sensu* Jordan & Rothschild (c’est-à-dire, femelle ayant un sinus net sur le sternite VII, mâle montrant un télomère à marge distale arrondie, portant trois soies) est donc cerné au nord et au sud de son aire de répartition connue par deux populations dont le sternite VII des femelles ne montre aucune incisure; si les mâles de ces populations se différencient, les femelles ne semblent pas séparables les unes des autres.

## Description de nouvelles sous-espèces

### *Nosopsyllus (N.) incisus traubi* Beaucournu & Laudisoit n. ssp. (figure 6)

#### Matériel de description

*Cf*. ci-dessus “Éthiopie, province de Bale”. Holotype ♂ sur *Dendromus* sp., Dinshu, Parc National, Monts Bale, 3 017 m, 21 février 1973; Allotype ♀ *ex nido Dendromus* sp., même endroit, même date; tous les autres exemplaires sont considérés comme paratypes (R. Traub & J. Ash *rec.*).

Dépôt des types : au CMNH (Pittsburg, USA), à l’exclusion d’une paire de paratypes conservée dans les collections du premier auteur.

*Derivatio nominis* : cette population est dédiée à R. Traub qui, avec J. Ash, récolta ces exemplaires.

#### Description sommaire

Assez proche de la forme nominative dont elle se séparera par les caractères qui suivent.

Mâles : segment IX : télomère large, arrondi sur son bord distal; la longueur du bord dorsal, de l’épine antéro-marginale à l’insertion de la première forte soie marginale, est plus longue ou égale à l’espace séparant l’insertion de ces trois soies. Sternite montrant à la base du bras distal trois ou quatre soies courtes (par rapport à celles, longues, de *N. atsbi*) mais fortes et sclérotisées; sur la partie apicale de ce bras, la sétation est représentée par des micro-épines et non des micro-soies (sauf chez un spécimen). *Hamulus* étroit, long à bords parallèles (comme chez la sous-espèce suivante), et non en “bec” court comme chez *N. i. incisus* J. & R., sans convexité ventrale.

Femelles non séparables de celles de *N. atsbi*, ni de celles de la sous-espèce suivante : sternite VII non incisé, montrant seulement un lobe, plus ou moins marqué, surplombant une concavité subventrale.

Dimensions : holotype, 3,4 mm; allotype, 3,5 mm.

De même que *N. atsbi*, cette espèce est relativement grande.

#### Matériel de description

*Cf*. ci-dessus “Zambie et Malawi (North Rhodesia)”. Holotype ♂ sur *Aethomys* sp., Nyika Plateau (Malawi), *ca* 2 225 m, 14 décembre1963; allotype ♀ sur *Grammomys dolichurus surdaster*, même endroit, 30 août 1962 (I.F. Keymer *rec.*); tous les autres exemplaires sont considérés comme Paratypes.

Dépôt des types : au BMNH (London, UK) à l’exclusion d’une paire de paratypes conservée dans les collections du premier auteur.

*Derivatio nominis* : ces populations sont nommées en hommage à R.E. Lewis qui, le premier, émit un doute sur l’unicité de *N. incisus*.

#### Description sommaire

Proche de la forme nominative dont elle se séparera par les caractères qui suivent.

Mâles : tergite VIII grand, à contour anguleux (cette forme peut se retrouver en Tanzanie). Segment IX : télomère nettement triangulaire, ses trois soies regroupées dans l’angle supéro-postérieur ou à proximité immédiate. *Hamulus* étroit, long à bords parallèles (comme chez *N. i. traubi*), et non en bec court comme chez *N. i. incisus* J. & R. Sur le dessin, nous avons représenté l’*hamulus* d’un paratype, celui de l’holotype étant masqué.

Femelles apparemment non séparables de celles de *N. atsbi*, ni de celles de *N. i. traubi* n. ssp.

Dimensions : holotype : 2,1 mm; allotype : 2,9 mm. C’est une taille classique pour un *Nosopsyllus* s. sto.
